# Humoral Immune Response after COVID-19 mRNA Vaccination in Patients with Liver Cirrhosis: A Prospective Real-Life Single Center Study

**DOI:** 10.3390/biomedicines11051320

**Published:** 2023-04-28

**Authors:** Elisa Biliotti, Alessandro Caioli, Chiara Sorace, Raffaella Lionetti, Eugenia Milozzi, Chiara Taibi, Ubaldo Visco Comandini, Fabrizio Maggi, Vincenzo Puro, Gianpiero D’Offizi

**Affiliations:** 1 Infectious Diseases Hepatology Department, National Institute for Infectious Diseases Lazzaro Spallanzani IRCCS, 00149 Rome, Italy; 2 Infectious Disease Clinic, Policlinico Tor Vergata, 00133 Rome, Italy; 3 Laboratory of Virology, National Institute for Infectious Diseases Lazzaro Spallanzani IRCCS, 00149 Rome, Italy; 4 Risk Management Unit, National Institute for Infectious Diseases Lazzaro Spallanzani IRCCS, 00149 Rome, Italy

**Keywords:** COVID-19, cirrhotic patients, mRNA vaccination, humoral response, male sex, past HCV infection, anti-spike antibodies titres

## Abstract

Coronavirus-disease-2019 (COVID-19) mRNA vaccination effectively reduces mortality and morbidity in cirrhotic patients, but the immunogenicity and safety of vaccination have been partially characterized. The study aimed to evaluate humoral response, predictive factors, and safety of mRNA-COVID-19 vaccination in cirrhotic patients compared to healthy subjects. A prospective, single-center, observational study enrolled consecutive cirrhotic patients who underwent mRNA-COVID-19 vaccination from April to May 2021. Anti-spike-protein (anti-S) and nucleocapsid-protein (anti-N) antibodies were evaluated before the first (T0) and the second (T1) doses and 15 days after completing the vaccination. An age and sex-matched healthy reference group was included. The incidence of adverse events (AEs) was assessed. In total, 162 cirrhotic patients were enrolled, 13 were excluded due to previous SARS-CoV-2 infection; therefore, 149 patients and 149 Health Care Workers (HCWs) were included in the analysis. The seroconversion rate was similar in cirrhotic patients and HCWs at T1 (92.5% vs. 95.3%, *p* = 0.44) and T2 (100% in both groups). At T2, anti-S-titres were significantly higher in cirrhotic patients compared to HCWs (2776.6 vs. 1756 BAU/mL, *p* < 0.001]. Male sex (*β* = −0.32 [−0.64, −0.04], *p* = 0.027) and past-HCV-infection (*β* = −0.31 [−0.59, −0.04], *p* = 0.029) were independent predictors of lower anti-S-titres on multiple-gamma-regression-analysis. No severe AEs occurred. The COVID-19-mRNA vaccination induces a high immunization rate and anti-S-titres in cirrhotic patients. Male sex and past-HCV infection are associated with lower anti-S-titres. The COVID-19-mRNA vaccination is safe.

## 1. Introduction

Cirrhotic patients have increased morbidity and mortality due to liver-related and non-liver-related complications of coronavirus disease 2019 (COVID-19) caused by the Severe Acute Respiratory Syndrome Coronavirus 2 (SARS-CoV-2) infection, compared to the general population [1,2,3]. A mortality rate up to 45% has been described in patients with decompensated cirrhosis [4]. Accordingly, the European Association for the Study of the Liver (EASL) and American Association for the Study of Liver Diseases (AASLD) guidelines recommended prioritizing these patients for COVID-19 vaccination [5,6], which is the most effective measure to reduce hospitalization rates, major complications and deaths [7,8,9]. Nevertheless, there are limited data on COVID-19 vaccines among patients with cirrhosis due to the limited number of cirrhotic patients enrolled in phase III clinical studies. Indeed, less than 0.1% of participants with advanced liver disease and 0.6% of participants with no better-defined liver disease received the BNT162b2 (Pfizer-BioNTech) and mRNA-1273 (Moderna) mRNA vaccines in the registrative trials, respectively [7,8].

The evaluation of COVID-19 vaccine immune response is important in subjects with cirrhosis since they have alterations in the immune system and vaccine hypo-responsiveness that has been observed with pneumococcal, hepatitis B, and influenza vaccines [10,11,12].

A few studies have specifically explored COVID-19 vaccine response in cirrhotic patients and reported conflicting results, probably due to differences regarding the stage of cirrhosis of enrolled subjects, the type of vaccine used, the duration of post-vaccination follow-up and, in a certain number of studies, the lack of a healthy control group [13]. Collectively, this highlights the scarcity of data on vaccine efficacy, immune responses, and their predictive factors in cirrhotic patients who have received COVID-19 vaccines.

We conducted an observational single-center prospective study to evaluate the humoral response, its associated predictive factors, and the safety of the COVID-19 mRNA vaccine in patients with cirrhosis compared to healthy subjects.

## 2. Materials and Methods

This prospective, single-center, observational study enrolled consecutive patients with liver cirrhosis of any etiology followed at the Hepatology Unit of National Institute for Infectious Diseases Lazzaro Spallanzani who agreed to receive COVID-19 mRNA vaccination from April 2021 to May 2021.

The study design also included a reference group of healthcare workers (HCWs), matched for age and sex, who received COVID-19 mRNA vaccines in January 2021.

Exclusion criteria included age < 18 years, inability to provide informed consent, and previous SARS-CoV-2 infection, defined by a positive nasopharyngeal swab for SARS-CoV-2 before enrolment or a positive result at baseline for antibodies against SARS-CoV-2 nucleocapsid protein (anti-N).

The primary endpoint of the study was to evaluate the humoral response to COVID-19 mRNA vaccines, measured as SARS-CoV-2 anti-spike protein antibodies (anti-S) titers, after 15 days from the second vaccine dose compared to HCWs.

The secondary endpoints were to identify predictors of humoral response to COVID-19 mRNA vaccines after 15 days from the second vaccine dose and to assess the safety of vaccination, defined by the incidence of adverse events, following the first and the second dose of vaccination.

Clinical characteristics (age, sex, BMI), etiology of liver disease, liver and non-liver-related comorbidities, and concomitant medications of all patients were collected from clinical records. Laboratory data up to three months prior to the administration of the first dose of COVID-19 vaccination (total bilirubin, aspartate aminotransferase (AST), alanine aminotransferase (ALT), albumin, creatinine, sodium, and international normalized ratio (INR) were also collected from clinical records. Child–Pugh and Model for End-Stage Liver Disease (MELD-sodium) scores were calculated in each enrolled patient. The estimated glomerular filtration rate (e-GFR) was calculated with the CKD-EPI 2019 formula. The presence of cirrhosis was confirmed using clinical or biochemical evidence (splenomegaly, ascites, hepatic encephalopathy), transient elastography, liver imaging, endoscopy, or liver biopsy.

Patients were vaccinated with the COVID-19 mRNA-1273 (Moderna) vaccine according to the standard protocol, with 28 days between the first and the second dose. Blood samples to evaluate humoral response were taken before the first dose of vaccine (T0), before the second dose of vaccine (T1), and 15 days after completing vaccination (T2). The reference group of HCWs had received COVID-19 mRNA-1273 (Moderna) or BNT162b2 (Pfizer-BioNTech) vaccines according to the standard protocol with 28 and 21 days between the first and the second dose, respectively. Humoral response to vaccination had been detected in the reference group at the same time points (T0, T1, and T2).

The potential adverse events (AEs) of COVID-19 vaccination were collected using a questionnaire that each patient filled out 7 days following the first and the second doses. The following adverse events were reported: local injection site reaction, including pain, swelling, redness, axillary swelling, and tenderness on the vaccination arm, and systemic reactions, including fatigue, headache, muscle pain, chills, joint pain, fever, and nausea/vomiting. AEs were reported according to the Common Terminology Criteria for AEs (CTCAE) grade scale.

The study was approved by the National Institute for Infectious Diseases Lazzaro Spallanzani Ethics Committee (Roma, Italy, Protocol Number 3580). All subjects gave their written consent to participate in the study, which was carried out in conformity with the 2013 revision of the Declaration of Helsinki, and written informed consent was obtained from each individual recruited for the study.

### 2.1. Humoral Response

The anti-S titers were quantified by a chemiluminescence microparticle antibody assay (ARCHITECT® i2000sr Abbott Diagnostics enzyme immunoassay) that tests for antibodies against the receptor-binding domain (RBD) of the SARS-CoV-2 spike protein. According to the manufacturer’s instructions and to the cut-off used in a previously published study [14,15], individuals with anti-S antibody concentrations ≥ 7.1 binding antibody units per milliliter (BAU/mL) were considered responders to the vaccine.

The anti-N antibody titers were evaluated by a chemiluminescence microparticle antibody assay (ARCHITECT SARS-CoV-2 IgG, Abbott Laboratories) and quantified at the same time-points to either confirm or detect SARS-CoV-2 infections.

### 2.2. Statistical Analysis

Data are displayed as median (interquartile range) for continuous variables and as absolute number (percentage) for categorical variables.

Continuous variables were compared between the two groups by the *t*-test or the Mann–Whitney U-test, according to the distribution of the variable (evaluated via Q-Q plots and Kolmogorov–Smirnov test). Comparisons between categorical variables were analyzed by the Chi-Square test of independence or Fisher’s Exact test.

The 149 sex and age-matched HCWs were selected from a larger group of 559 SARS-CoV-2 naive HCWs, using 1:1 nearest neighbor propensity score matching without replacement, with propensity scores obtained from a probit regression model [16].

To identify independent variables that were associated with lower humoral immunological response (anti-S titers), regression analyses were performed using generalized gamma models with a log link function.

Residual analysis for these models was conducted using the DHARMa package [17], which implements a simulation-based approach to obtain quantile residuals from the fitted model [18,19].

A gamma regression model on the whole study group (149 patients with cirrhosis and 149 HCWs), with covariates sex, age, presence of cirrhosis, and the type of vaccine, was used to estimate the effect of cirrhosis on the anti-S titers at T2.

On the cirrhotic group, univariable gamma regression was performed on the anti-S titers at T2 for a list of candidate predictors. Variables that were significantly associated with the serologic response (*p* < 0.05) were included in the multiple regression model, together with other known predictors of serologic response (age and sex) and variables deemed clinically relevant for our sample of patients (Child–Pugh score and immunosuppressive treatment).

Non-parametric bootstrap resampling was performed to validate the confidence intervals for the coefficients obtained from the multiple regression model. The model was fit again on 1000 bootstrapped samples, and the resulting empirical cumulative distribution functions of the coefficients estimates were used to assess the 95% confidence intervals.

R software version 4.1.2 [20] was used to perform the statistical analysis.

## 3. Results

### 3.1. Patient Characteristics

One hundred and sixty-two consecutive cirrhotic patients were enrolled in the study between April 2021 and May 2021. Thirteen subjects were excluded from the study since they had a history of SARS-CoV-2 infection and/or tested positive for anti-N antibodies at baseline (T0). Therefore, 149 cirrhotic patients and 149 age and sex-matched HCWs were included in the analysis.

All cirrhotic patients received the COVID-19 mRNA-1273 vaccine (Moderna) according to the standard protocol with 28 days between the first and the second dose. The reference group of HCWs received the COVID-19 BNT162b2 vaccine (Pfizer-BioNTech) in 147 cases (98.7%) and the mRNA-1273 vaccine (Moderna) in 2 cases (1.3%). The median age of HCWs was 57 years [IQR, 51–61 years], and the prevalence of male sex was 65.1%.

The demographic and clinical characteristics of cirrhotic patients are shown in Table 1.

The median age was 60.0 years (IQR, 55.0–64.0 years), the majority of patients were males (71.1%), the median BMI was 26.1 Kg/m^2^ (IQR, 23.8–29 Kg/m^2^). Ninety-one patients (61.1%) showed at least one comorbidity; the most frequent concomitant diseases were arterial hypertension (18.8%), history of malignancies (16.8%), and diabetes (13.4%).

One hundred and one patients (67.8%) showed a unique etiology of liver disease, while in 44 patients (29.5%), two or more etiological factors were ascertained, and in 4 patients (2.7%), the etiology of cirrhosis was cryptogenic. The most common etiology of cirrhosis was past hepatitis C virus (HCV) infection (64.4%, n = 96), followed by alcoholic liver disease (ALD) (27.5%, n = 41), hepatitis B virus (HBV) infection (20.1%, n = 30), autoimmune hepatitis (3.0%, n = 5), primary biliary cholangitis (6.0%, n = 9), hepatitis B and D (HBV/HDV) coinfection (4.7%, n = 7), cryptogenic (2.7%, n = 4), nonalcoholic steatohepatitis (NASH) (2.7%, n = 4) and biliary atresia (0.7%, n = 1).

The majority of patients had compensated cirrhosis (Child-Pugh A5 or A6) (89.3%, n = 133), while 10.7% (n = 16) had decompensated cirrhosis (Child-Pugh B or C). The median MELD-Na score was 9.7 (IQR, 7.24–12.06). The median e-GFR was 96.0 mL/min/1.73 m^2^ (IQR, 82.2–103.1 mL/min/1.73/m^2^).

Liver-related complications affected 47% (n = 70) of participants. The presence of esophageal varices was the most common complication, affecting 30.2% (n = 45) of our sample, while hepatocellular carcinoma (HCC) and ascites affected respectively 20.8% (n = 31) and 9.4% (n = 14) of participants.

Ten (6.7%) patients were on immunosuppressive therapy. In eight patients, the treatment consisted of a monotherapy regimen with either azathioprine (3 patients), glucocorticoid (3 patients), cyclosporine (1 patient), or regorafenib (1 patient). In two patients, immunosuppressive treatment consisted of two medications: prednisone associated with tacrolimus in one case and with azathioprine in the other case.

### 3.2. Antibody Response after COVID-19 Vaccination

After the first vaccine dose, the positive rate of anti-S antibodies was 92.5% (135/146) in tested patients with cirrhosis compared to 95.3% (142/149) in the reference group (*p* = 0.44). After the second vaccine dose, the positive rate of anti-S antibodies was 100% (149/149) in both cirrhotic patients and HCWs.

At T1, the median anti-S titers were significantly higher in patients with cirrhosis compared to HCWs (117 [42.7–265.2] vs. 73.7 [41–122] BAU/mL, *p* = 0.004). At T2, the median anti-S titers remained significantly higher in the study group compared to HCWs (2776.6 [1463.2–4820] vs. 1756 [1062–2878] BAU/mL, *p* < 0.001). Since all patients with cirrhosis had been vaccinated with COVID-19 mRNA-1273 (Moderna), while nearly all HCWs had been vaccinated with COVID-19 BNT162b2 (Pfizer-BioNTech), a further analysis was carried out. A gamma regression analysis performed in the whole study group (149 patients with cirrhosis and 149 HCWs) and including age, sex, the presence of cirrhosis, and the type of vaccine (mRNA-1273 or BNT162b2), showed that the presence of cirrhosis tended to be associated with lower anti-S titers at T2 even if the result was not statistically significant (β = −0.39 [−1.71, 0.54], *p* = 0.48) (Supplementary Table S1).

Among patients with cirrhosis, univariable and multivariable analyses of factors associated with anti-S titers after the second dose of the COVID-19 vaccine are shown in Table 2.

On univariable gamma regression, male sex (β = −0.32 [−0.61, −0.04], *p* = 0.03) and HCV etiology (β = −0.36 [−0.63, −0.09], *p* = 0.01) were found to be independent baseline predictors of lower anti-S titers after the second dose of vaccination.

**Figure 1:**
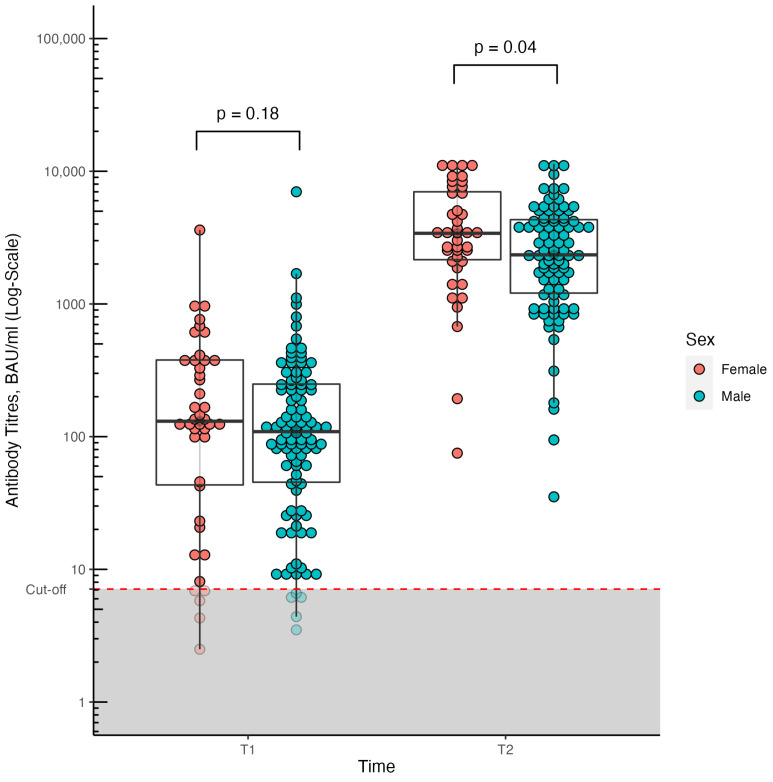
Humoral response (anti-S titers) to COVID-19 vaccination at different time points (T1 and T2) in patients with cirrhosis stratified according to sex (male vs. female). Concentrations above 7.1 BAU/mL were considered positive, and concentrations below 7.1 BAU/mL as negative.

At T2, the median anti-S titers were significantly lower in male compared to female patients with cirrhosis (2425 [1230–4364] vs. 3447 [2183–6918] BAU/mL, *p* = 0.04), while at T1 both seroconversion rate (90.4% vs. 93.3%, *p* = 0.73) and median anti-S titers (106 [42.3–242] vs. 131 [43.3–378] BAU/mL, *p* = 0.18) were comparable (Figure 1).

**Figure 2:**
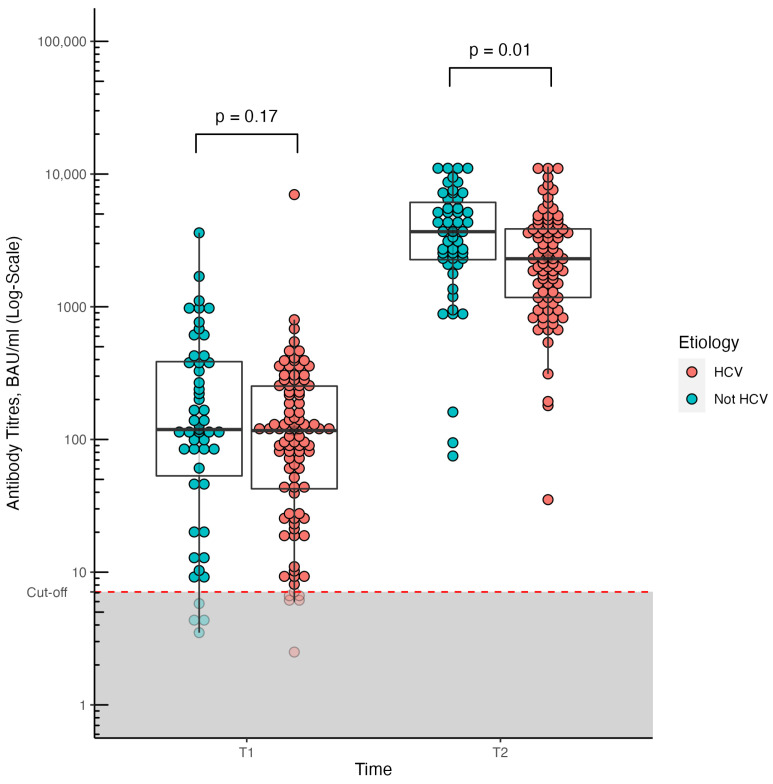
Humoral response (anti-S titers) to COVID-19 vaccination at different time points (T1 and T2) in patients with cirrhosis stratified according to the etiology of liver disease (HCV vs. other types). Concentrations above 7.1 BAU/mL were considered positive, and concentrations below 7.1 BAU/mL as negative.

At T2 patients with HCV-related cirrhosis showed significantly lower median anti-S titers compared to patients with other etiologies of liver disease (2304 [1194–3939] vs. 3691 [2266–6401] BAU/mL, *p* = 0.005), while at T1 both seroconversion rate (91.7% vs. 88.7%, *p* ≈ 1) and median anti-S titers (116 [34–240] vs. 119 [53.6–386] BAU/mL, *p* = 0.17) were comparable (Figure 2).

On multiple gamma regression, after adjusting for age, Child–Pugh score, and concurrent immunosuppressive therapy, both male sex (β = −0.34 [−0.64, −0.04], *p* = 0.03) and HCV etiology of liver disease (β = −0.31 [−0.59, −0.04], *p* = 0.03) remained statistically significant (Table 2).

At T2, both the presence of Child–Pugh B or C cirrhosis and immunosuppressive treatment were not found to be predictors of lower anti-S titers on univariable and multivariable regression analysis (Table 2).

Patients with Child–Pugh B or C cirrhosis had a slightly but not statistically significant lower seroconversion rate at T1 compared to patients with Child–Pugh A cirrhosis (87.5% vs. 93.1%, *p* = 0.34), but median anti-S titers were similar between the two groups (101 [37.3–129] vs. 118 [43.4–277] BAU/mL, *p* = 0.31). At T2 median anti-S titers remained similar in both groups (2481 [1771–5443] BAU/mL in Child–Pugh B or C cirrhotics vs. 2796 [1459–4562] BAU/mL in Child–Pugh A cirrhotics, *p* = 0.62). Patients under immunosuppressive therapy had a slightly but not statistically significant lower seroconversion rate at T1 compared to other patients (80% vs. 93.4%, *p* = 0.16), with comparable median anti-S titers both at T1 (91.4 [10.1–245] vs. 117 [44.6–260] BAU/mL, *p* = 0.59) and T2 (2112 [1212–4192] vs. 2830 [1558–4843] BAU/mL, *p* = 0.42).

### 3.3. Vaccine Safety

The m-1273 COVID-19 vaccine (Moderna) showed a good safety profile since no serious and unexpected adverse events were reported in patients with cirrhosis.

At least one local side effect was experienced by 61.7% of the patients after the first or the second vaccine dose. The most common local side effects were pain (61.1%), swell (10.7%), and redness (8.7%) at the injection site.

At least one systemic side effect was experienced by 31.5% of the patients. The most common systemic side effects were fatigue (24.8%), headache (9.4%), fever (8.7%), arthralgia (7.4%), and myalgia (6.7%).

Both local and systemic side effects were transient and self-limiting (Table 3).

## 4. Discussion

The present study describes the humoral response of 149 patients with cirrhosis after a standard protocol-based vaccination of two doses of the mRNA-based COVID-19 vaccine m-1273 (Moderna). In our study, the response rate to vaccination of cirrhotic patients was encouraging since the seroconversion rate 15 days after the second dose was 100% and comparable to the HCWs.

Currently, data on the effectiveness of COVID-19 vaccination rely on post-approval real-world studies due to the limited inclusion of participants with liver diseases in phase III clinical trials. A recent meta-analysis including seven observational studies that evaluated humoral immune response (anti-S antibody) after two doses of COVID-19 vaccination in subjects with chronic liver disease reported an overall seroconversion rate of 91% (95% CI, 83–95%) and of 85% (95%CI, 75–91%) in the subgroup of cirrhotic patients [13].

Published studies analyzing immunogenicity of mRNA COVID-19 vaccine in patients with advanced chronic liver disease showed better results with seroconversion rates ranging from 94.1% to 100% in 725 patients with liver cirrhosis enrolled in 7 real-world studies [21,22,23,24,25,26,27]. Therefore, the cirrhosis-associated immune dysfunction does not seem to affect the humoral response to mRNA COVID-19 vaccination, suggesting that mRNA vaccination may represent a promising alternative to conventional vaccine approaches in this setting of patients.

In the present study, after the first and second doses of mRNA COVID-19 vaccination, the median titers of anti-S antibodies were significantly higher in cirrhotic patients compared to HCWs. We speculate that this surprising result could be due to the different types of mRNA vaccine used in cirrhotic patients (Moderna, 1273 mRNA vaccine) and in the HCWs reference group (Pfizer-BioNTech, BNT162b2 mRNA vaccine). Our hypothesis is supported by previously published studies where the Moderna-1273 mRNA vaccine induced significantly higher anti-S titers compared to other types of vaccination in the general population [28,29,30] and in immunocompromised subjects [31,32]. In addition, two studies evaluating the effectiveness of COVID-19 vaccination in cirrhotic patients demonstrated that a single dose of Johnson & Johnson vaccine [21] or two doses of BNT162b2 mRNA vaccine [24] were associated to a suboptimal antibody response. The differences in the titers of anti-S antibodies may be potentially due to the higher mRNA content in 1273-mRNA compared with the BNT162b2 mRNA vaccine (100 vs. 30 µg, respectively) and the longer interval between the first and the second dose for the 1273-mRNA (4 weeks) compared with BNT162b2 vaccine (3 weeks). It is important to underline that these studies are not randomized; therefore, further data are needed to clarify the role of the type of vaccine in the effectiveness of vaccination in patients with advanced chronic liver disease.

In the present study, both the presence of Child-Pugh class B/C cirrhosis and immunosuppressive treatment were not found to be predictors of lower anti-S titers after two doses of 1273-mRNA vaccination on univariable and multivariable regression analysis. Although Child-Pugh class B/C cirrhosis was underrepresented among the enrolled patients, accounting for 10.1% and 0.7% respectively, the result agrees with previously published studies that used an mRNA COVID-19 vaccine and included a larger number of decompensated cirrhotics [21,22,27]. Regarding the role of immunosuppressive treatment on the humoral response to mRNA COVID-19 vaccination, two studies confirmed our result [23,24], while the study by Thuluvath and colleagues demonstrated that two but not one immunosuppressive medication was independently associated with a poor antibody response [21] and the study by Bakasis and colleagues found that immunosuppressive treatment was negatively correlated with anti-S antibody titers and neutralizing activity [26]. In our study, only ten patients were on immunosuppressive treatment, and the majority of them (80%) took only one medication; this could explain why immunosuppressive treatment did not result in a predictor of lower serological response to COVID-19 vaccination.

In our study, HCV-related cirrhosis was an independent risk factor for a lower serological response after two doses of mRNA COVID-19 vaccination (β = −0.31 [−0.59, −0.04], *p* = 0.03), although all enrolled subjects were sustained virologic responders to antiviral treatment. The possible role of the etiology of liver disease on the humoral response to COVID-19 vaccination has been poorly investigated. Two Chinese studies evaluated the antibody response in 381 patients with nonalcoholic fatty liver disease (NAFLD) and in 362 patients with chronic hepatitis B at least 14 days after the second dose of the virus-inactivated whole virion SARS-CoV-2 vaccine, the seroconversion rate was 95.5% and 97.8% respectively and was similar to healthy individuals [33,34]. Among the other published studies, the different etiology of liver diseases (viral, NAFLD, NASH, ALD, autoimmune hepatitis) are represented in widely distinct proportions, and there are no studies enrolling only patients with HCV-related liver disease. A lower immunogenicity of HBV vaccination in HCV patients compared to the general population has been reported in some studies [35,36,37], which persisted in subjects who achieved SVR following interferon-based therapy [38]. Several studies demonstrate that patients with chronic hepatitis C have an impaired adaptive immunity with dysfunctional CD4+ T cells, CD8+ T cells and natural killer (NK) cells, atypical memory B cells, and diminished mucosal-associated invariant T (MAIT) cell compartment [39]. Research on the reversal of immunity in patients that are cured from a long-lasting HCV infection found that many imprints of chronic HCV infection on distinct immune compartments persist for years despite direct-acting antiviral treatments [39,40] and may be responsible for the finding of our study. Unfortunately, we did not perform characterization of the immune response, such as analyzing the memory B-cell and T-cell-mediated immune reaction or the neutralizing capacity of antibodies; therefore, definitive conclusions on this topic can not be achieved, but represent an interesting aspect for further studies.

Finally, our study suggested that the male sex is an independent risk factor for a lower serological response after two doses of mRNA COVID-19 vaccination (β = −0.34 [−0.64, −0.04], *p* = 0.03). The majority of studies assessing humoral response to COVID-19 vaccination in cirrhotic patients did not demonstrate the sex-related difference, although a Chinese study, including 437 patients with chronic liver disease who received two doses of inactivated whole virion SARS-CoV-2 vaccine, reported that male sex was an independent risk factor for negative serological response to vaccination (OR 1.89; 95% CI 1.12–3.90; *p* = 0.017) [41]. In addition, this result is consistent with published studies that demonstrated that the male sex is implicated in a poor humoral response to COVID-19 vaccination in the mouse model [42], in hospitalized patients with liver dysfunction [43] and in HCWs [44], on the contrary female sex is associated with higher post mRNA COVID-19 vaccination antibody titers in the general population [45]. In general, the finding of our study is not surprising since it is well established that females induce stronger immune functions and higher antibody levels, composed of more functional antibodies, compared to males and also experience more adverse reactions to vaccination. Some of these differences can be linked to hormonal differences, particularly to the estrogen level, but additional factors, such as miRNAs or other genetic/epigenetic differences between males and females, could influence humoral immunity [46].

Lastly, several studies demonstrated the safety of mRNA COVID-19 vaccination in cirrhotic patients [21,22,23,24,25,26,27]. Our study confirms that the schedule (two doses) of mRNA-1273 (Moderna) vaccination is safe in cirrhotic patients since no serious safety signals were reported.

There are some limitations to our study: (1) The study group and the reference group were administered different types of mRNA COVID-19 vaccine, respectively 1273 mRNA (Moderna) and BNT162b2 mRNA (Pfizer/BioNTech); (2) we measured anti-S antibodies only, although their titer has been shown to correlate very well with that of neutralizing antibodies [44]; (3) additional characterization of the immune response, such as analyzing the memory B-cell and T-cell-mediated immune reaction, could be of interest and is not represented in this study; (4) the follow-up period is short (15 days after completing vaccination); (5) monocentric cohort; (6) relatively small sample size of the Child–Pugh score B/C cirrhosis subgroup.

## 5. Conclusions

This study demonstrates that COVID-19 mRNA vaccination induces a surprisingly high rate of immunization and anti-S antibodies titers in both compensated and decompensated cirrhotic patients. Male sex and past HCV infection were the factors associated with poorer serologic response. The study also confirms the safety of the mRNA COVID-19 vaccine in patients with advanced chronic liver diseases.

## Figures and Tables

**Table 1:** Baseline characteristics of the 149 patients with cirrhosis.

	Whole Sample (n = 149)	Child-Pugh A (n = 133)	Child-Pugh B/C (n = 16)
**Demographic Characteristics**			
Age, years	60 (55–64)	60 (55–64)	60 (56–62.5)
Male sex	106 (71.14)	92 (69.17)	14 (87.5)
BMI, Kg/m^2^	26.15 (23.78–29)	26 (23.8–28.5)	29.94 (23.85–34.35)
**Laboratory parameters**			
e-GFR, mL/min/1.73 m^2^	96.02 (82.23–103.07)	94.81 (81.16–102.58)	101.71 (97.58–108.94)
Total bilirubin, mg/dL	0.93 (0.7–1.36)	0.9 (0.7–1.23)	2.26 (1.91–3.07)
INR	1.1 (1.01–1.2)	1.09 (1–1.17)	1.27 (1.19–1.47)
ALT, U/L	24 (16–38.75)	24 (16–37.5)	33 (18.5–43)
AST, U/L	29 (22–41.75)	28 (22–40.5)	39 (27.5–65)
**Comorbidities**			
Any comorbidity	91 (61.07)	81 (60.9)	10 (62.5)
Diabetes	20 (13.42)	17 (12.78)	3 (18.75)
COPD	11 (7.38)	8 (6.02)	3 (18.75)
Arterial Hypertension	28 (18.79)	27 (20.3)	1 (6.25)
HIV infection	10 (6.71)	10 (7.52)	0 (0.0)
History of Myocardial Infarction	4 (2.68)	4 (3.01)	0 (0.0)
History of malignancies	25 (16.78)	23 (17.29)	2 (12.5)
Other	57 (38.26)	51 (38.35)	6 (37.5)
**Etiology of Cirrhosis**			
HCV	96 (64.43)	86 (64.66)	10 (62.5)
HBV	30 (20.13)	28 (21.05)	2 (12.5)
HBV+HDV	7 (4.7)	7 (5.26)	0 (0.0)
ALD	41 (27.52)	31 (23.31)	10 (62.5)
AILD	5 (3.36)	5 (3.76)	0 (0.0)
PBC	9 (6.04)	9 (6.77)	0 (0.0)
BA	1 (0.67)	1 (0.75)	0 (0.0)
NASH	4 (2.68)	4 (3.01)	0 (0.0)
Cryptogenic	4 (2.68)	4 (3.01)	0 (0.0)
**Cirrhosis Staging Scores**			
CP A	133 (89.26)	133 (100.0)	0 (0.0)
CP B	15 (10.07)	0 (0.0)	15 (93.75)
CP C	1 (0.67)	0 (0.0)	1 (6.25)
MELD-Na score	9.7 (7.72–12.27)	9.31 (7.52–11.26)	14.34 (13.28–16.86)
**Complications of Cirrhosis**			
Any complication	70 (46.98)	56 (42.11)	14 (87.5)
Hepatocellular carcinoma	31 (20.81)	26 (19.55)	5 (31.25)
Esophageal varices	45 (30.2)	33 (24.81)	12 (75)
Ascites	14 (9.4)	6 (4.51)	8 (50)
**Therapy**			
Immunosuppressive therapy	10 (6.71)	10 (7.52)	0 (0.0)

BMI, Body Mass Index; e-GFR, Estimated Glomerular Filtration Rate; INR, International Normalized Ratio; COPD, Chronic Obstructive Pulmonary Disease; MELD, Model for End-Stage Liver Disease; ALD, Alcholic Liver Disease; AILD, Autoimmune Liver Disease; PBC, Primary Biliary Cirrhosis; BA, Biliary Atresia; NASH, Non-Alcoholic Steatohepatitis.

**Table 2:** Univariable and Multivariable regression analysis to predict the lower humoral response (anti-S titers) after two doses of the COVID-19 vaccine in 149 patients with cirrhosis.

		Univariable Analysis		Multivariable Analysis		
	Category	Coefficient (95% CI)	*p*	Coefficient (95% CI)	*p*	Validated Coefficients
Age, years	Continuous	−0.01 (−0.02, 0.01)	0.3105	−0.01 (−0.02, 0.01)	0.4301	−0.006 (−0.019, 0.008)
Sex	Male vs. Female	**−0.32 (−0.61, −0.04)**	**0.0283**	**−0.34 (−0.64, −0.04)**	**0.027**	**−0.339 (−0.606, −0.041)**
BMI, Kg/m^2^	Continuous	0 (0, 0.02)	0.3425			
One or more comorbidities	Yes vs. No	0.09 (−0.19, 0.36)	0.5384			
Two or more comorbidities	Yes vs. No	−0.15 (−0.44, 0.14)	0.2963			
Three or more comorbidities	Yes vs. No	−0.21 (−0.62, 0.26)	0.3492			
Child-Pugh score	B/C vs. A	0.19 (−0.24, 0.67)	0.4146	0.23 (−0.19, 0.7)	0.3084	−0.43 (−1.088, 0.129)
MELD-Na	Continuous	0.01 (−0.03, 0.05)	0.5666			
e-GFR, mL/min/1.73 m^2^	Continuous	0 (0, 0.01)	0.5339			
Immunosuppressive Therapy	Yes vs. No	−0.15 (−0.65, 0.43)	0.5756	−0.4 (−0.92, 0.19)	0.1444	0.221 (−0.368, 0.719)
HCV	Yes vs. No	**−0.36 (−0.63, −0.09)**	**0.0108**	**−0.31 (−0.59, −0.04)**	**0.0287**	**−0.314 (−0.594, −0.007)**
HBV	Yes vs. No	−0.11 (−0.42, 0.22)	0.5083			
HBV+HDV	Yes vs. No	−0.06 (−0.61, 0.61)	0.855			
ALD	Yes vs. No	0.27 (−0.02, 0.57)	0.0753			
AILD	Yes vs. No	0.24 (−0.22, 0.77)	0.3348			
NASH	Yes vs. No	0.02 (−0.69, 0.92)	0.9632			
HIV	Yes vs. No	−0.17 (−0.65, 0.37)	0.502			
Hepatocellular carcinoma	Yes vs. No	−0.07 (−0.39, 0.26)	0.6585			

**Table 3:** Adverse reactions (AEs) after either dose of the 1273-mRNA vaccine among the 149 patients with cirrhosis.

	Whole Sample (n = 149)	Child–Pugh A (n = 133)	Child–Pugh B/C (n = 16)
Any AE	101 (67.79)	91 (68.42)	10 (62.5)
**Local Adverse Events**			
Any local AE	92 (61.74)	83 (62.41)	9 (56.25)
Pain	91 (61.07)	82 (61.65)	9 (56.25)
Redness	13 (8.72)	11 (8.27)	2 (12.5)
Swelling	16 (10.74)	16 (12.03)	0 (0.0)
Lymphadenopathy	1 (0.67)	1 (0.75)	0 (0.0)
**Systemic Adverse Events**			
Any systemic AE	47 (31.54)	42 (31.58)	5 (31.25)
Fever	13 (8.72)	13 (9.77)	0 (0.0)
Fatigue	37 (24.83)	32 (24.06)	5 (31.25)
Headache	14 (9.4)	13 (9.77)	1 (6.25)
Myalgia	10 (6.71)	10 (7.52)	0 (0.0)
Arthralgia	11 (7.38)	11 (8.27)	0 (0.0)
Nausea	0 (0.0)	0 (0.0)	0 (0.0)
Vomiting	0 (0.0)	0 (0.0)	0 (0.0)
Diarrhea	1 (0.67)	1 (0.75)	0 (0.0)

## Data Availability

Not applicable.

## Supplementary Materials

The following supporting information can be downloaded at: https://www.mdpi.com/article/10.3390/biomedicines11051320/s1.

## Abbreviations

The following abbreviations are used in this manuscript:

COVID-19	Coronavirus disease 2019
Anti-S	Anti-spike protein
Anti-N	Anti-nucleocapsid protein
AEs	Adverse Events
HCWs	Health Care Workers
SARS-CoV-2	Severe Acute Respiratory Syndrome Coronavirus 2
EASL	European Association for the Study of the Liver
AASLD	American Association for the Study of Liver Diseases
BMI	Body Mass Index
e-GFR	Estimated Glomerular Filtration Rate
INR	International Normalized Ratio
COPD	Chronic Obstructive Pulmonary Disease
MELD	Model for End-Stage Liver Disease
ALD	Alcoholic Liver Disease
AILD	Autoimmune Liver Disease
PBC	Primary Biliary Cirrhosis
BA	Biliary Atresia
NASH	Non-Alcoholic Steatohepatitis
AST	Aspartate amino-transferase
ALT	Alanine amino-transferase
MELD	Model for End-Stage Liver Disease
INR	International Normalized Ratio
RBD	Receptor Binding Domain
HCC	Hepatocellular Carcinoma
