# Supplementary material for: Humoral Immune Response after COVID-19 mRNA Vaccination in Patients with Liver Cirrhosis: A Prospective Real-Life Single Center Study
Source: Biomedicines. 2023 Apr 28;11(5):1320. doi: 10.3390/biomedicines11051320 (PMC10215518; doi:10.3390/biomedicines11051320)
Supplement: Supplementary file 1 [file biomedicines-11-01320-s001.zip › biomedicines-2334134-supplementary.pdf]

	Category	Coefficient (95% CI)	P
Sex	Male vs. Female	**-0.27 (-0.47, -0.08)**	**0.008**
Age	Continuous	-0.01 (-0.02, 0)	0.0559
Cirrhosis	Yes vs. No	-0.39 (-1.71, 0.54)	0.4872
Vaccine	Moderna vs. Pfizer	0.92 (-0.01, 2.25)	0.0978
